# Mendelian Randomization Validates the Immune Landscape Mediated by Aggrephagy in Esophageal Squamous Cell Carcinoma Patients from the Perspectives of Multi-omics

**DOI:** 10.7150/jca.93376

**Published:** 2024-02-12

**Authors:** Haiyang Yu, Gao Si, Fuchun Si

**Affiliations:** 1 Traditional Chinese Medicine (Zhong Jing) school, Henan University of Chinese Medicine, Zhengzhou 450046, China.; 2 Henan Key Laboratory of TCM Syndrome and Prescription Signaling, Henan International Joint Laboratory of TCM Syndrome and Prescription Signaling, Academy of Chinese Medical Sciences, Henan University of Chinese Medicine, Zhengzhou 450046, China.; 3 Department of Orthopedic, The Third Hospital of Peking University, Beijing 100029, China.

**Keywords:** Mendelian Randomization, Single-cell, Multi-omics, Esophageal Cancer, Causal Relationships

## Abstract

**Objective:** To delineate the immune landscape of ESCC patients mediated by aggrephagy through bioinformatics and identify prognostic cell cluster genes with causal attributes to esophageal cancer through Mendelian randomization.

**Methods:** Quality control, dimension reduction, and annotation were performed on the ESCC single-cell dataset. NMF clustering of various cell subgroups was carried out based on the expression of AGG-related genes, and AGG-related genes in each cluster were identified. Pseudo-temporal analysis was used to observe changes in the expression of AGG-related genes in each cluster. Cell communication analysis was employed to observe interactions between cell subgroups. Changes in classification, metabolism, or KEGG pathways in related subgroups were observed based on different cell characteristics. The AGG cluster attributes of TCGA and GEO samples were assessed based on GSVA, and the prognosis of each cluster was observed. The immune treatment situation and the relationship between mutation level and prognosis of AGG cluster-related samples were observed through the TIDE database and microsatellite instability. Finally, the eQTL of genes in each prognostic AGG cluster was used as an instrumental variable, with esophageal cancer as the outcome factor. Through Mendelian randomization analysis, AGG cluster-related genes with a causal relationship to esophageal cancer were established.

**Results:** Dimension reduction clustering of single-cell transcriptome data identified 19 different cell subgroups. After re-annotation of the 19 cell subgroups, it was found that the CAF cells, B cells, T cells, NK cells, etc., of ESCA patients were all elevated compared to the control group. CAF cells had a high degree of communication with most cells. There were significant differences in macrophage metabolism and B-cell-mediated signal transduction pathways in different AGG clusters. The TUBA1B+Mac-C0 cluster, along with other clusters, exhibits predictive prognostic and immunotherapeutic potential at the transcriptional level. Mendelian randomization analysis revealed a causal relationship between genes such as CTSZ, CTSC, DAD, COLEC12, ATOX1, within the AGG cluster, and the onset of esophageal cancer.

**Conclusion:** Aggrephagy mediates and influences the alterations and interactions of various immune cells in patients with ESCC. We elucidate the roles of AGG-related clusters, such as TUBA1B+Mac-C0, VIM+CD8+T_cells-C0, UBB+Mac-C2, in mediating prognosis and immune therapy in ESCC patients. Genes causally associated with the occurrence of esophageal cancer are identified within the AGG cluster, including CTSZ, CTSC, DAD, COLEC12, ATOX1, etc., offering new evidence for clinical immune therapy. These findings underscore the significance of these gene clusters in influencing both prognosis and immune responses in the context of esophageal cancer, shedding light on potential therapeutic targets and prognostic markers.

## Introduction

Esophageal squamous cell carcinoma (ESCC) represents a significant subtype of esophageal cancer^1^. With its symptoms often concealed, late clinical manifestation, and rapid progression, the 5-year survival rate for ESCC patients is a mere 30% to 40%^2^. Current treatments for esophageal cancer primarily involve traditional surgical removal, radiation, and chemotherapy, but these interventions have limited efficacy and severe side effects^3^. This is associated with the poor immunogenicity of ESCC and its complex immunosuppressive microenvironment. Numerous studies have suggested a potential link between the tumor's immune microenvironment and patient prognosis. Therefore, to accurately predict the molecular mechanisms and prognosis of ESCC patients, it is imperative to explore new and effective biomarkers for early diagnosis and accurate prognosis prediction of ESCC.

Aggrephagy (AGG), a type of selective autophagy, is a critical pathway for cells to clear large amounts of misfolded proteins^4^. Protein aggregates are regulated by phase separation during their formation, transitioning between various states such as liquid and solid, which influences the nature of the aggregates and their clearance methods^5^. The formation of protein aggregates can generate new protein toxicities, which, in addition to losing their normal functions, can interact with other properly folded proteins, causing intracellular protein function disorder. Studies have shown that protein aggregates can affect various diseases, including cancer and cardiovascular diseases^6^. Clearing intracellular protein aggregates can assist in restoring normal cellular physiological functions and is an important way to delay disease progression^7^. It is certain that AGG mediates the effects of various immune cells on the occurrence and development of tumors in patients with ESCC through synergistic or opposing actions, but the specific roles and molecular mechanisms are still unclear. Therefore, how to analyze the clinical significance of the tumor microenvironment mediated by aggrephagy has become a challenge for researchers.

Single-cell sequencing technology reveals the highly complex cellular composition of tumor tissues with high resolution, making it a powerful tool for studying tumor heterogeneity and the interactions among various cell groups^8^. Mendelian randomization (MR) is a newly emerged method for inferring causal effects. This method uses genetic variation as an instrumental variable, based on the strong association between the instrumental variable and the exposure factor to explore the causal relationship between exposure and outcome^9^. In this article, through a joint analysis of single-cell transcriptome data and transcriptomes, we focus on the molecular actions mediated by AGG-related genes in different cell subgroups of ESCC, and identify prognosis-related expression quantitative trait genes (EQTL) through Mendelian randomization, aiming to provide new insights for the precision treatment of ESCC.

## 1. Methodology

### Data Acquisition and Processing

Single-cell transcriptomic data were sourced from the GSE196756 dataset in the GEO database. Data were read using R and the Seurat package. Cell quality control criteria included^10^: (1) Cells expressing <300 genes. (2) UMI (Unique Molecular Identifiers) of mitochondrial genome >50%. (3) UMI of ribosomal genome <3%. Genes from the mitochondrial genome, housekeeping gene MALAT1, and those detected in fewer than three cells were removed. Normalization and other processing were conducted following standard procedures, and the R package Harmony was used to integrate metastatic and primary cancer samples^11^. Transcriptomic data were obtained from the public databases The Cancer Genome Atlas (TCGA, https://cancergenome.nih.gov/) and GEO database (https://www.ncbi.nlm.nih.gov/geo). The TCGA database contained 160 cases of esophageal cancer tissue, 11 cases of normal esophageal tissue, and 183 cases of related clinical data were downloaded, including age, clinical stage, tumor grade, and survival status. The GSE53625 dataset in the GEO database contained mRNA transcription data for 129 normal and 129 tumor tissues.

### Cell Annotation and Clustering

The Seurat package was used for quality control of single-cell data. Principal component analysis (PCA) was used for dimension reduction, followed by secondary dimension reduction using the uniform manifold approximation and projection (UMAP) algorithm. Based on the PCA dimension reduction results, UMAP was used for visualization of single-cell clustering, and the t-distributed stochastic neighbor embedding (t-SNE) clustering algorithm was used to obtain subdivided cell clusters. Cells were annotated based on cell marker genes from the literature. Subgroup cells were processed following the Seurat standard procedure. Immune cell clustering mainly referred to the report by Zhang Zemin's team^12^-^14^, and CAF (cancer-associated fibroblast) clustering referred to the research by Elyada and others and NuRmik and others^15^,^16^. AGG-related genes were sourced from The Molecular Signatures Database (MSigDB, https://www.gsea-msigdb.org/)^17^.

### Feature Analysis of Cell Subgroups

Based on the expression of AGG-related genes, the NMF package was used for dimension reduction and clustering of each cell group^18^. The expression of Maker genes was used to further identify related subgroups within each cell group. Specific cells were extracted for NMF clustering and AGG clustering. The Monocle R package was used for pseudo-temporal analysis and single-cell differentiation trajectory analysis of AGG genes in each cell group^19^. SCENIC was used to explore the expression of transcription factors in cells^20^. The ClusterProfilerR software package was used for gene ontology (GO) functional enrichment analysis and Kyoto encyclopedia of genes and genomes (KEGG) pathway enrichment analysis of differential gene sets. CellChat was used for analysis of cell communication. This software identifies overexpressed receptor pairs and constructs a PPI network based on gene expression in single-cell transcriptome sequencing, calculates communication probabilities, and infers the communication network of cell interactions^21^. Heatmaps were used to display the number and intensity of interactions between cells. The classification, metabolism, or changes in KEGG pathways of related subgroups were observed based on the characteristics of different cells.

### Construction and Verification of Prognostic Models

Gene set variation analysis (GSVA) was employed to analyze the transcriptional differences of AGG-related genes between ESCC patients and normal individuals. We extracted marker genes for the AGG clusters defined in our single-cell transcriptome analysis. Single-sample enrichment analysis through GSVA was conducted to obtain AGG-related cluster scores for each transcriptome sample. Discrepancies in each AGG cluster between ESCC patients and normal samples were observed. The surv_cutpoint function from the survminer package was utilized for optimal cutpoint determination and visualization of continuous variables in survival data. The TCGA dataset served as the training set, while the GEO dataset was used for validation^22^. Single-factor COX regression analysis was performed to assess the impact of each AGG cluster on the prognosis of esophageal cancer patients, with visualization using the ggplot2 package.

### Evaluation of Immunotherapy and Microsatellites

Jiang P and others developed the Tumor immune dysfunction and exclusion (TIDE) algorithm (http://tide.dfci.harvard.edu) by integrating two mechanisms of tumor immune evasion^23^. A lower TIDE score indicates a better response to immunotherapy. We conducted an analysis of the relationship between GSVA scores of different AGG clusters in patients and their response to immunotherapy. Logistic regression analysis was performed on the results from TCGA and GEO datasets to observe the consistency between the two datasets. The impact of different AGG clusters on disease prognosis was validated using the IMvigor210 cohort.

### Mendelian Randomization Verification of Key Gene eQTLs

Maker genes with prognostic difference clusters were extracted, and related eQTLs were extracted from the IEU Open GWAS project database (https://gwas.mrcieu.ac.uk/datasets/) as exposure factors^24^. The esophageal cancer queue also came from this database, with the ID ieu-b-4960, which contained whole genome data of 372,016 normal samples and 740 esophageal cancer samples, which were used as outcome data. Mendelian randomization analysis was conducted on the eQTL of maker genes in each cluster and esophageal cancer to find out the maker genes with causal relationship with esophageal cancer in each cluster. In our study, there was no sample overlap between populations, and all participants belonged to the European population, mitigating potential bias due to racial differences. When using Maker genes as the exposure factor^25^, we initially selected SNPs that were strongly correlated with gene expression and reached genome-wide significance at P < 5×10^-8^. Subsequently, we computed the F-statistic to assess the strength of the association between the instrumental variable and the exposure factor, using the formula F = (beta/se)^2^, where beta represents the allelic effect size, and se denotes the standard error^26^. SNPs with F-values less than 10 were excluded to address potential weak instrumental variable bias^27^. The inverse variance-weighted fixed-effects model (IVW-FE) was employed as the primary Mendelian randomization (MR) analysis method. Cochran's Q test was applied to assess heterogeneity among instrumental variables, with P > 0.05 suggesting minimal likelihood of heterogeneity. The MR Egger intercept test was conducted to evaluate horizontal pleiotropy, and if the intercept term was statistically significant, it indicated substantial horizontal pleiotropy. Finally, genes exhibiting both heterogeneity and pleiotropy were excluded to identify prognostic genes causally related to esophageal cancer. Subsequently, GeneMANIA database (http://genemania.org/) and the enrichplot package were utilized for protein-protein interaction (PPI) and Gene Ontology (GO) analyses.

## Results

### Processing of Single-Cell Transcriptome Data

**Figure 1:**
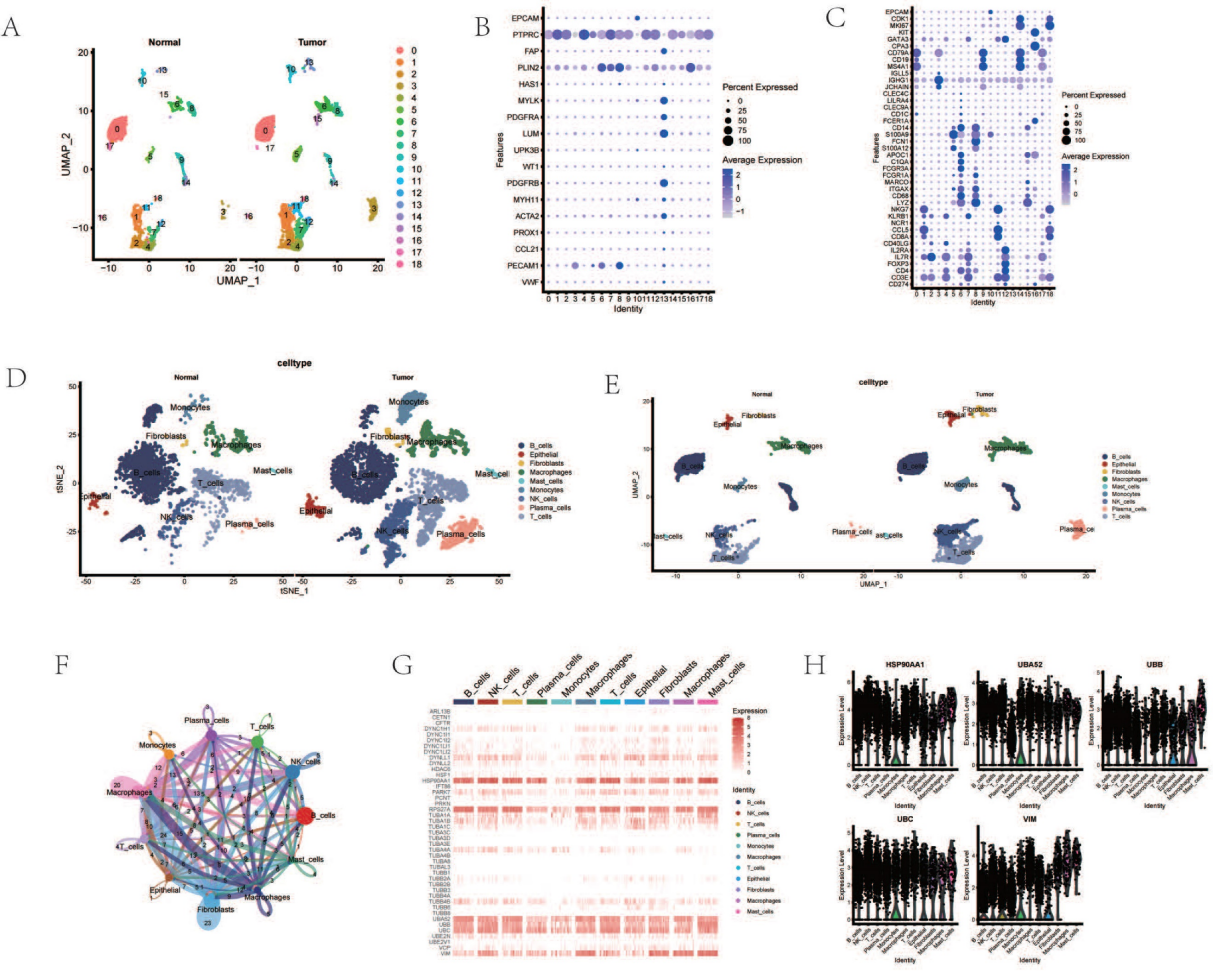
Processing of Single-Cell Transcriptome Data. A. Dimensionality reduction clustering of ESCA samples. B. Expression of tissue cell marker genes. C. Expression of immune cell marker genes. D. Cell annotation tSNE clustering. E. Cell annotation UMAP clustering. F. Cell communication. G. Heatmap of AGG gene expression in different cells. H. Expression of AGG high expression genes in different cells.

Dimensionality reduction and clustering of single-cell transcriptomic data identified 19 distinct cellular subgroups (Figure 1 A). These 19 cell subgroups were re-annotated based on the expression of marker genes for immune cells and other cells (Figure 1 B, C). Upon re-annotation of these 19 cell subgroups, it was found that in ESCA patients, the levels of CAF cells, B cells, T cells, NK cells, and other cell subgroups were elevated compared to the control group (Figure 1 D, E). Cell communication results revealed a high degree of communication between CAF cells and the majority of other cells, suggesting that fibroblasts may interact with various immune cells (Figure 1 F). Genes such as HSP90AA1, UBA52, RPS27A, TUBB4B, UBB, UBC, and VIM were generally highly expressed in various cell subgroups (Figure 1 G). Interestingly, the enrichment and expression strength of these genes differed among different cell subgroups. For instance, most genes such as HSP90AA1, UBA52, and UBB were enriched in B cells, NK cells, and T cells, yet the expression levels of genes such as VIM and UBB were stronger in fibroblasts, macrophages, and adipocytes (Figure 1 H).

### Analysis of Cancer Associated Fibroblasts (CAFs)

**Figure 2:**
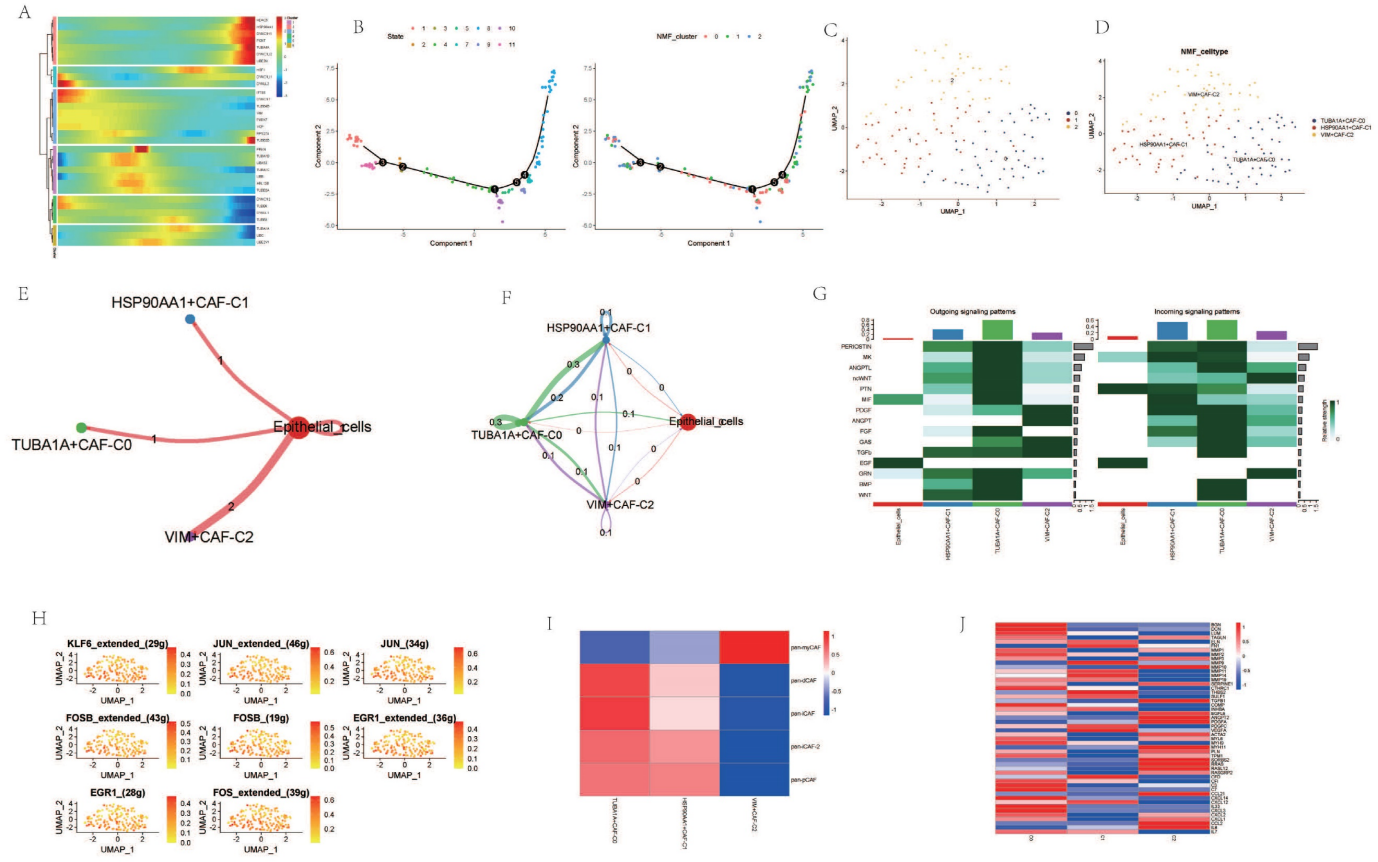
Analysis of Cancer Associated Fibroblasts (CAFs). A. Pseudo-temporal analysis of AGG gene in CAF cells. B. Cellular trajectory of CAF cells. C. NMF clustering of CAF cells based on the expression of the AGG gene. D. Annotation of AGG clusters after dimension reduction of CAF cells. E. Communication between AGG clusters and Epithelial cells. F. Global communication between AGG clusters and Epithelial cells. G. Receptor-related interaction strength between AGG clusters and Epithelial cells. H. Distribution of transcription factors in CAF cell subtypes. I. CAF subtypes in AGG clusters. J. Expression of immune microenvironment-related genes in AGG clusters.

Cancer Associated Fibroblasts (CAFs) are considered one of the most abundant stromal cells in all types of solid tumors, associated with a series of pro-tumorigenic biological processes such as tumor cell invasion, cancer stem cell renewal, chemotherapy resistance, and immune cell evasion^28^-^31^. Dimensionality reduction of fibroblast subgroups followed by pseudotemporal analysis revealed temporal differences in the expression levels of AGG-related genes (Figure 2 A). Cell trajectory analysis simulated the differentiation trends of different clusters (Figure 2 B). Based on the expression of AGG-related genes, fibroblasts were clustered using NMF. Three new clusters were identified based on the expression of AGG-related genes in each cluster, namely TUBA1A-CAF-C0, HSP90AA1-CAF-C1, and VIM-CAF-C1. Cell communication analysis showed that all three clusters had strong interactions with epithelial cells (Figure 2 E, F). Multiple co-receptors such as PTN, MIT, EGF, etc., also showed varying levels of expression in epithelial cells and the three clusters (Figure 2 G). Transcription factors such as KLF6, JUN, FOSB, etc., also had different expressions in the three clusters (Figure 2 H). Comparing the expression of different CAF subgroup maker genes revealed that VIM-CAF-C1 cluster only had higher expression in pan-myCAFs. Both TUBA1A-CAF-C0 and HSP90AA1-CAF-C1 clusters were distributed in other CAF subtypes, especially the genes related to TUBA1A-CAF-C0 cluster showed stronger expression in non-pan-myCAFs subtypes, indicating a more pronounced manifestation of its other CAF subtypes (Figure 2 I). In addition, different clusters mediated different TME genes, which could potentially mediate the occurrence of different tumor microenvironments (Figure 2 J).

### Analysis Related to CD8+ Cells

**Figure 3:**
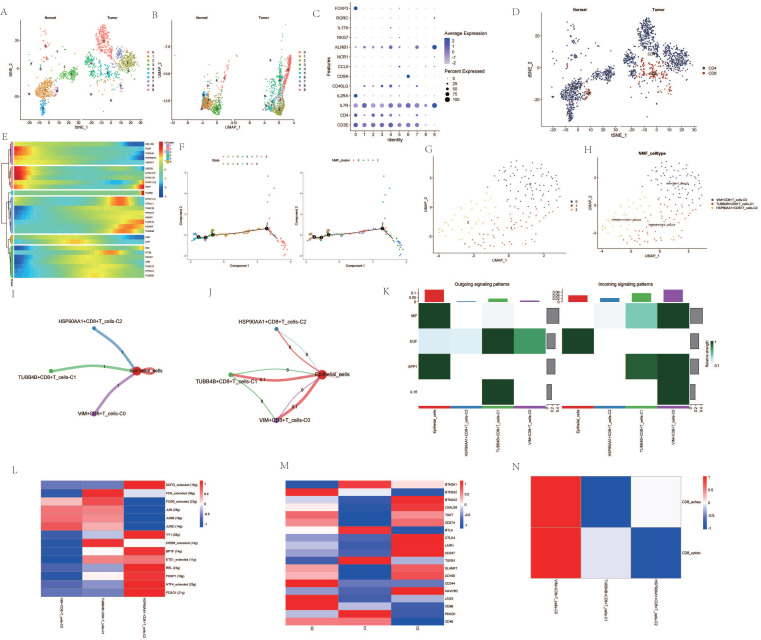
Analysis Related to CD8+ Cells. A. t-SNE dimensionality reduction clustering of T cells. B. UMAP dimensionality reduction clustering of T cells. C. Marker genes of T cells. D. Subgroup annotation of T cells. E. Pseudo-temporal analysis of AGG genes in CD8+ cells. F. Cellular trajectory of CD8+ cells. G. NMF clustering of CD8+ cells based on the expression of AGG genes. H. Annotation of AGG clusters after dimensionality reduction of CD8+ cells. I. Communication between AGG clusters and Epithelial cells. J. Global communication between AGG clusters and Epithelial cells. K. Interaction intensity related to receptor pairing between AGG clusters and Epithelial cells. L. Distribution of transcription factors in AGG clusters. M. Distribution of TME genes in AGG clusters. N. Types of T cells in AGG clusters.

Upon dimensionality reduction of T cells, a significant variation in the number of T cell clusters was observed between cancer patients and normal individuals (Figure 3A, B). After extracting and annotating the marker genes of different T cell subgroups, it was observed that the subgroups of CD4 and CD8 cells in cancer patients were significantly elevated (Figure 3C, D). Pseudo-temporal analysis revealed notable expression changes in genes such as HSP90AA1, TUBA4A, PCNT, and VIM among CD8 cells at different stages of differentiation, and the differentiation of different clusters also showed a certain sequence (Figure 3E, F). After re-clustering the CD8 cell subgroups post-NMF clustering, VIM+CD8 T_cell-C0, TUBB4B+CD8+T_cell-C1, HSP90AA1+CD8+T_cell-C2 were identified (Figure 3G, H). Cellular communication analysis revealed that all three clusters had a low degree of interaction with epithelial cells, and the interaction among them was also weak (Figure 3I, J). However, the intensity of communication-related signals showed high and low expressions among different clusters (Figure 3K). Most transcription factors were expressed most highly in the HSP90AA1+CD8+T_cell-C2 cluster, but were less expressed in the VIM+CD8+T_cell-C0 cluster (Figure 3L). TME-related genes such as CTLA4, LAIR1, CD247 were also expressed more in the HSP90AA1+CD8+T_cell-C2 cluster, but were less expressed in the other two clusters (Figure 3M). Analysis of CD8 T cell subtypes showed that in the VIM+CD8+T_cell-C0 cluster, the proportion of exhausted T cells and cytotoxic T cells was relatively high, while other clusters did not show significant subtype characterization (Figure 3N).

### Analysis Related to Macrophages

**Figure 4:**
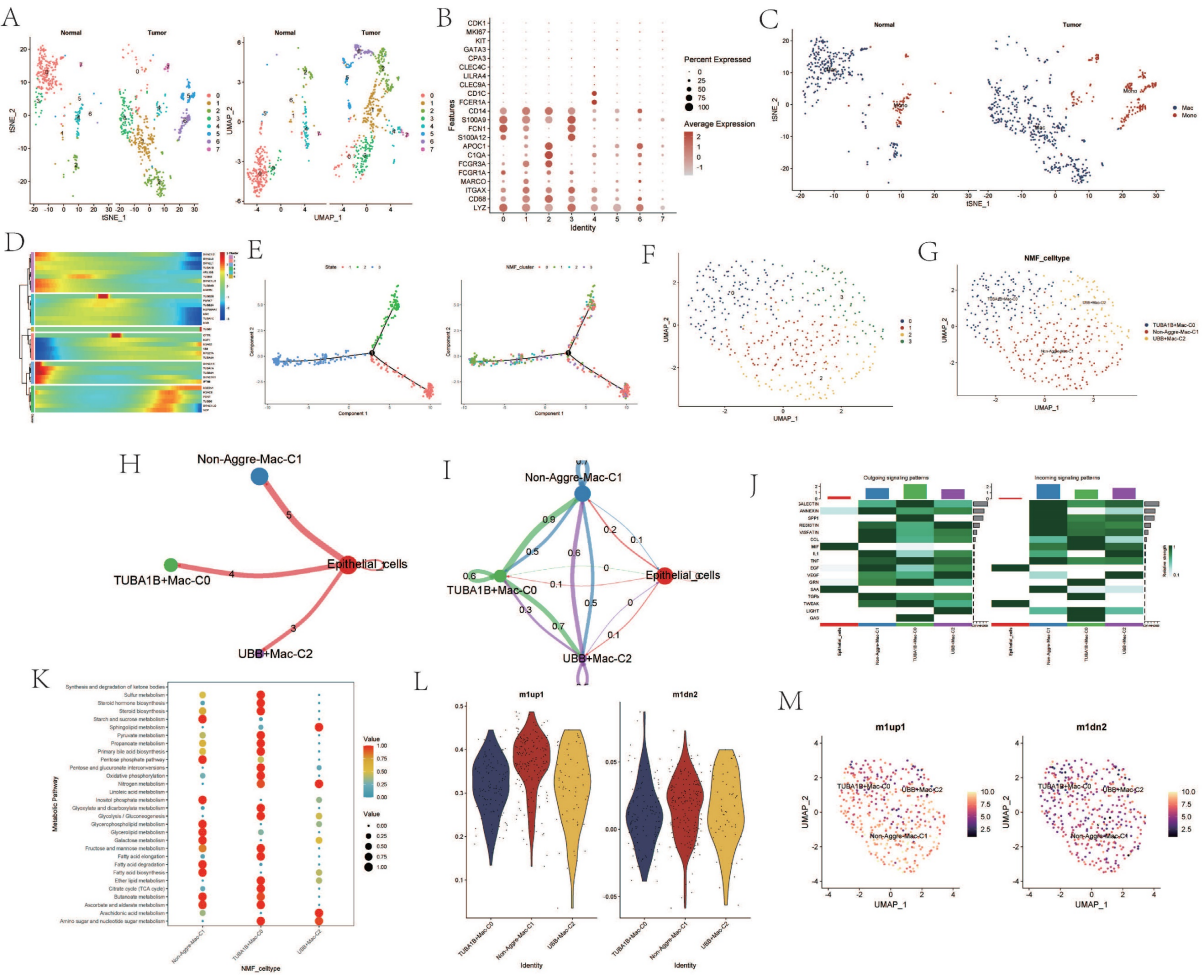
Analysis Related to Macrophages. A. tSNE and UMAP dimensionality reduction clustering of macrophages. B. Marker genes of macrophages. C. Subgroup annotation of macrophages. D. Pseudo-temporal analysis of AGG gene in macrophages. E. Cellular trajectory of macrophages. F. NMF clustering of macrophages based on the expression of AGG gene. G. Annotation of AGG cluster after dimensionality reduction of macrophages. H. Communication between AGG cluster and Epithelial cells. I. Global communication between AGG cluster and Epithelial cells. J. Interaction strength related to receptor pairing between AGG cluster and macrophages. K. Differences in metabolic pathways of macrophages in AGG cluster. L. Expression of marker genes of M1 and M2 macrophages in AGG cluster. M. Spatial distribution of M1 and M2 macrophages in AGG cluster.

Upon re-dimensioning and clustering of macrophages, a notable difference in the number of clusters between tumor samples and normal samples was observed (Figure 4A). The expression of maker cells within each cluster was re-annotated (Figure 4B). The clusters after dimension reduction were found to be divided into macrophages and monocytes (Figure 4C). Following NMF clustering of the macrophages, the macrophage population could be divided into TUBA1B+Mac-C0, Non-Aggre-Mac-C1, and UBB+Mac-C2 clusters (Figure 4F, G). Pseudo-time analysis revealed changes in the expression of the AGG gene at different times (Figure 4D). The cell trajectory diagram revealed the differentiation trend of macrophages (Figure 4E). Cell communication also showed direct interaction between the three macrophage clusters and epithelial cells, and network interaction among the four (Figure 4 H, I). There were significant changes in the outgoing and incoming signals of different clusters (Figure 4J). The cell metabolic pathway indicated that the TUBA1B+Mac-C0 cluster had a higher enrichment level in metabolic pathways such as Sulfur metabolism, Steroid hormone biosynthesis, and Steroid biosynthesis. The Non-Aggre-Mac-C1 had a higher enrichment level in metabolic pathways such as Glycerophospholipid metabolism, Glycerolipid metabolism, and Galactose metabolism. The UBB+Mac-C2 had a higher enrichment level in metabolic pathways such as Ether lipid metabolism, Citrate cycle (TCA cycle), and Butanoate metabolism (Figure 4K). Macrophage polarization analysis showed that the three types of macrophage clusters tended to polarize towards M1 type (Figure 4L, M).

### Analysis Related to B Cells

**Figure 5:**
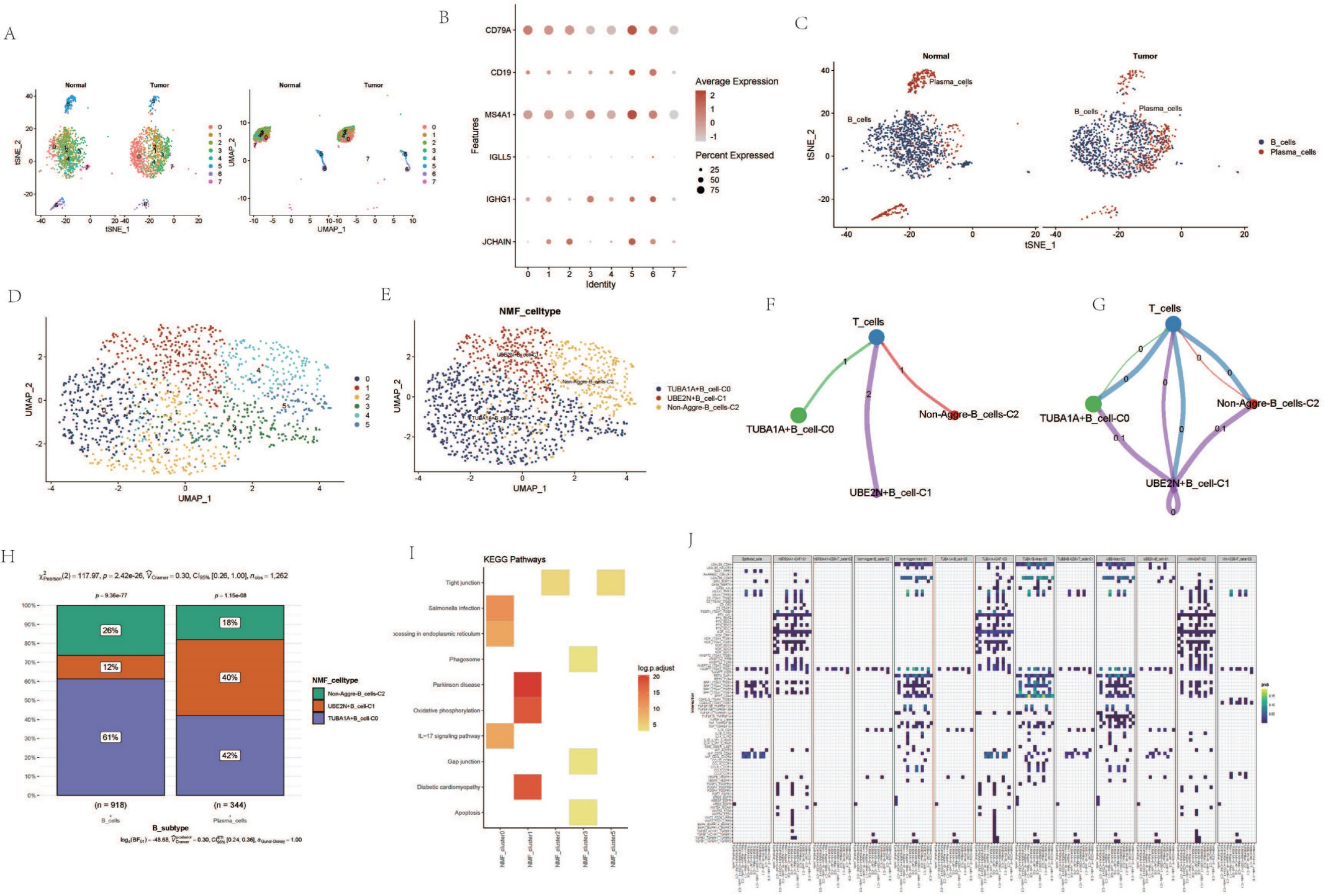
Analysis Related to B Cells. A. tSNE and UMAP dimensionality reduction clustering of B cells. B. Marker genes of B cells. C. Subgroup annotation of B cells. D. NMF clustering of B cells based on AGG gene expression. E. Annotation of AGG clusters after dimensionality reduction of B cells. F. Communication between AGG clusters and T cells. G. Global communication between AGG clusters and T cells. H. Proportion of each AGG cluster in B cells and plasma cells. I. Signal transduction mediated by AGG clusters in B cells. J. Global communication of AGG clusters in various cells.

Upon re-dimensioning the B-cell clusters, there appears to be no significant changes between the ESCA samples and the normal samples (Figure 5 A). B cell-related markers were extracted (Figure 5 B). Upon re-annotation of the re-dimensioned B cell cluster, a portion of plasma cells was identified (Figure 5 C). Following NMF clustering, new B cell clusters TUBA1A+B cell-C0, UBE2N+B cell-C1, and Non-Aggre-B_cells-C2 were identified (Figure 5 D, E). Cell communication showed direct interaction and potential association between the three B cell clusters and T cells. The proportions within different clusters of B cells and plasma cells varied, with the TUBA1A+B cell-C0 cluster having the highest proportion within B cells, and the UBE2N+B cell-C1 cluster having the lowest (Figure 5 F, G). In contrast, within plasma cells, the UBE2N+B cell-C1 cluster had the highest proportion and the TUBA1A+B cell-C0 cluster the lowest (Figure 5 H). Enrichment analysis revealed that TUBA1A+B cell-C0 was enriched in the IL-17 signaling pathway and Apoptosis pathway, UBE2N+B cell-C1 in the Oxidative phosphorylation pathway, and Non-Aggre-B_cells-C2 in the Tight junction pathway (Figure 5 I). Global cell communication analysis of all clusters and cell subgroups revealed high communication intensity between clusters such as HSP90AA1-CAF-C1, TUBA1A-CAF-C0, UBB+Mac-C2 and other subgroups (Figure 5 J).

### Prognostic Analysis of AGG Clusters at the Transcriptomic Level

**Figure 6:**
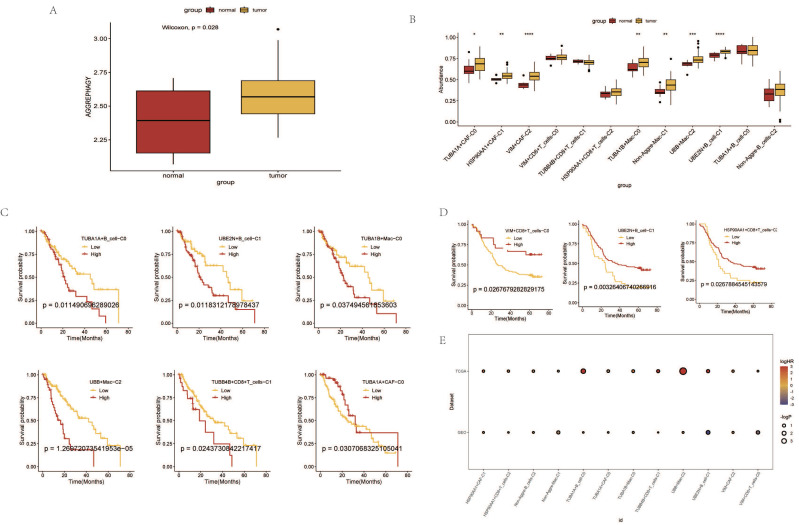
Prognostic Analysis of AGG Clusters at the Transcriptomic Level.A. Differential analysis of AGG gene set in TCGA via ssGSEA. B. Expression profiles of different AGG clusters in TCGA. C. Prognostic value of AGG clusters in TCGA. D. Prognostic value of AGG clusters in GEO. E. Prognostic risk bubble chart of AGG clusters in both TCGA and GEO.

Based on the expression levels of AGG-related genes in each sample from the TCGA data, a ssGSEA analysis was performed. The results indicated that the enrichment degree of AGG-related genes in ESCA patients was higher compared to normal samples (Figure 6 A). The expression of AGG genes in different clusters also shows significant differences from the normal group (Figure 6 B). A prognostic model is constructed based on the related genes of different clusters (Figure 6 C). It is found that the prognosis of 6 clusters in the TCGA dataset has significant differences. However, after verification in the GEO dataset, only 3 clusters have statistically significant prognosis, but the prognosis of UBE2N+B_cell-C1 cluster is opposite to that in the TCGA dataset (Figure 6 D). A bubble chart is constructed based on the results of univariate COX regression of the two datasets, and the prognostic effects of most clusters in the two datasets are largely consistent (Figure 6 E).

### Immune Response and Prognostic Analysis of AGG Clusters and Microsatellite Instability

**Figure 7:**
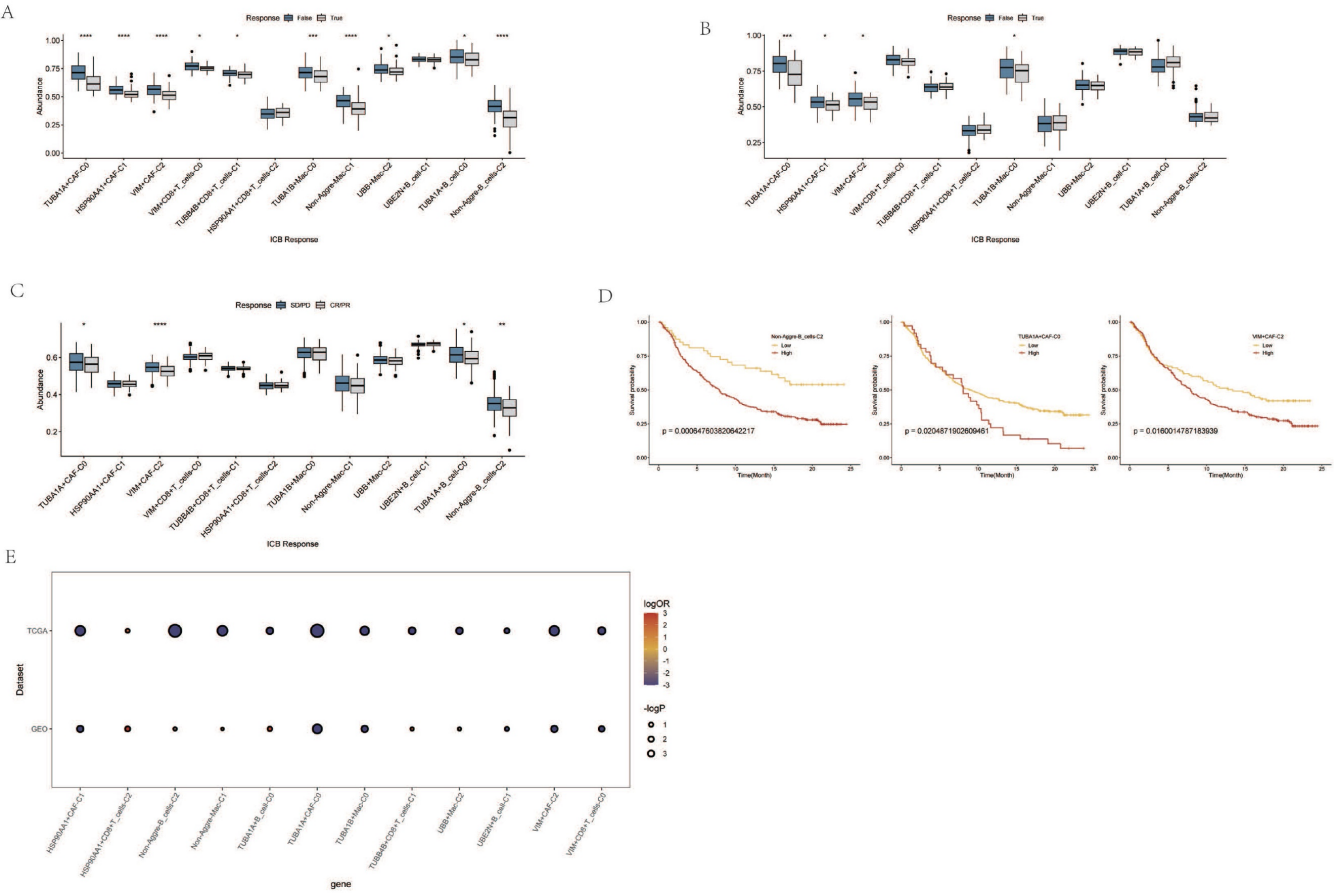
Immune Response and Prognostic Analysis of AGG Clusters and Microsatellite Instability. A. TIDE status of different AGG clusters in TCGA. B. TIDE status of different AGG clusters in GEO. C. Outcomes after immunotherapy of different AGG clusters. D. Prognostic differences of AGG clusters in microsatellite instability. E. Prognostic risk bubble chart of AGG clusters in TCGA and GEO.

Through the immune response analysis by TIDE, the effect of immunotherapy on each sample was predicted. The results revealed that the response of each cell cluster characteristic sample group to immunotherapy varied. Among them, clusters such as TUBA1A+CAF-C0, HSP90AA1+CAF-C1, VIM+CAF-C2, TUBA1B+Mac-C0 exhibited a lesser degree of sensitivity to immunotherapy in both TCGA and GEO samples (Figure 7A, B). There were also certain differences in the final outcomes of each cell cluster characteristic sample group in the TCGA database (Figure 7C). The prognostic risk bubble chart also showed that most clusters are risk factors for patients, often leading to unfavorable prognoses (Figure 7E). In the independent microsatellite cohort of bladder cancer, validation of our clusters revealed that the prognosis of patients in multiple clusters was better (Figure 7D). This suggests that our AGG gene set-associated cell clusters not only necessitate immunotherapy in esophageal cancer cohorts but also hold potential value for targeted therapy in other cancers.

### Mendelian Randomization Validates Key Gene's eQTL and Its Causal Relationship with Esophageal Cancer

Maker genes that reflect prognostic characteristics were extracted from the AGG clusters. Through Mendelian randomization of eQTL, it was found that the TUBA1B+Mac-C0 cluster and other cluster have the most maker genes with a causal relationship to esophageal cancer (Table 1).

**Figure 8:**
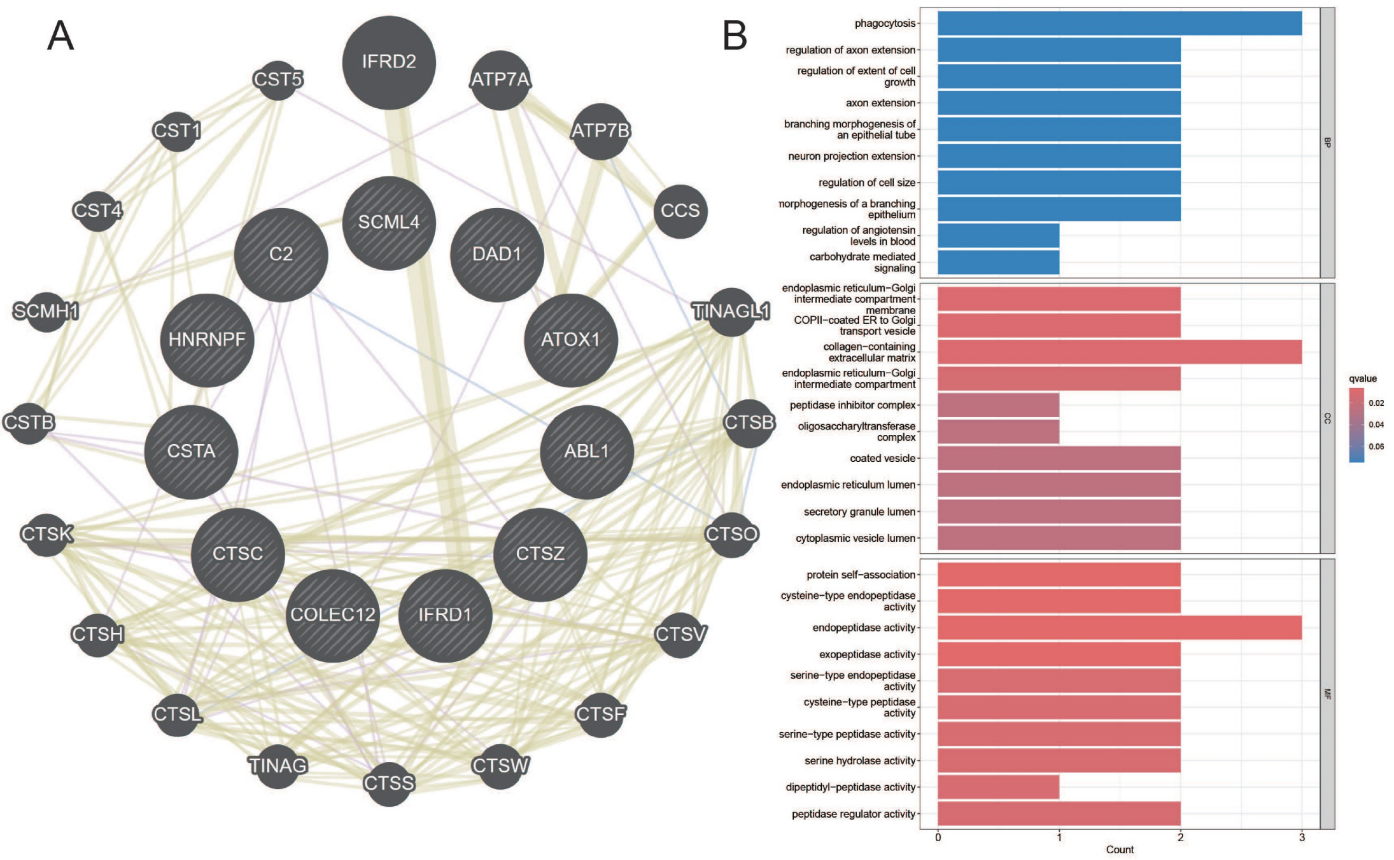
Co-expression Network and GO Enrichment Analysis of Causal Genes in AGG-Related Clusters. A. Co-expression network of causal genes in AGG-related clusters. B. GO enrichment analysis of causal genes in AGG-related clusters.

Among them, the genes CTSZ, CTSC, DAD, COLEC12, ATOX1, etc., in the TUBA1B+Mac-C0 cluster have a causal relationship with esophageal cancer. In the other clusters, the genes SCML4, HNRNPF, IFRD1, CSTA, ABL1, etc., have a causal relationship with esophageal cancer. The network diagram drawn clearly shows the related genes contained in each cluster (Figure 8A). The GO enrichment analysis revealed that these causal genes are predominantly associated with changes in cellular components such as the endoplasmic reticulum-Golgi intermediate compartment membrane, COPII-coated ER to Golgi transport vesicle, and collagen-containing extracellular matrix. Furthermore, they are related to molecular functions including protein self-association, cysteine-type endopeptidase activity, and endopeptidase activity (Figure 8B). This implies that changes in the expression of these genes can affect the occurrence of esophageal cancer.

## Discussion

Historically, our understanding of tumor initiation, progression, and metastasis has been largely derived from the genetic and phenotypic characteristics of tumor cells^32^. However, as research deepens, it has become evident that tumor development is inextricably linked to the tumor microenvironment, with both aspects influencing and promoting each other. Some scholars even propose that "cancer is a disease of the microenvironment and immunity." According to reports, changes in the immune microenvironment can lead to chronic inflammation in esophageal epithelial cells, thereby activating pro-inflammatory signaling pathways. Tumor cells can suppress the anti-tumor immune response by recruiting different immune cell populations in the microenvironment or expressing inhibitory molecules, enabling tumor cells to evade immune surveillance. Cell groups such as T cells, B cells, etc., promote immune escape of cancer cells by secreting cytokines and activating pro-inflammatory pathways, thereby facilitating the malignant progression of esophageal cancer^33^,^34^. CAFs contribute to tumor cell migration and invasion by secreting growth factors and altering the extracellular matrix, forming a tumor niche. Macrophages also exhibit other pro-tumor functions, including inducing blood vessel formation and promoting tumor cell invasion. It is important to note that changes in the immune microenvironment are often influenced by various signal transduction pathways. In this study, we employed Non-negative Matrix Factorization (NMF) clustering based on the expression of Aggrephagy-related genes to explore the interactions between cancer cells mediated by Aggrephagy and various immune cell subpopulations in esophageal cancer.

Interestingly, we found that the VIM-CAF-C1 cluster only had high expression on pan-myCAFs. Pan-myCAFs represent the main CAF subgroups present in many tumors and can promote cancer cell invasion^35^. VIM can drive epithelial-mesenchymal transition and fibroblast-to-myofibroblast transformation through the TGF-β/Smad signaling pathway, making it a common marker for fibroblasts. Consequently, the identified VIM-CAF-C1 cluster in our study may exhibit a higher invasiveness, given its association with fibroblasts and their potential role in promoting cellular transformations^36^,^37^. Cytotoxic T cells are the most potent influencers in anti-tumor immune responses, but when the effector function of T cells is low, they transform into an exhausted T cell phenotype. This is often manifested as a decrease in the ability of T cells to secrete cytokines, an increase in chemokine expression, and an increase in the expression of various inhibitory receptors such as PD-1, T cell immunoglobulin mucin domain 3 (TIM-3), lymphocyte-activation gene 3 (LAG3), CTLA-4, and T cell Ig and ITIM domain (TIGIT). In the VIM+CD8+T_cell-C0 cluster, both exhausted T cells and cytotoxic T cells showed an increasing trend. This suggests that the T cells in the VIM+CD8+T_cell-C0 cluster are more active and mediate intense cellular immunity in the body. Different macrophage clusters also have varying degrees of enrichment in cellular metabolic pathways. For example, the TUBA1B+Mac-C0 cluster shows high enrichment in metabolic pathways such as Sulfur metabolism, Steroid hormone biosynthesis, and Steroid biosynthesis^38^. The metabolism of these sulfur compounds in the body can promote various biological processes such as enzyme catalysis, energy transfer, and redox metabolism, thereby affecting tumor development. Steroid hormones have potent anti-inflammatory and immunosuppressive effects, as well as pleiotropic effects on innate and adaptive immune responses^39^. Non-Aggre-Mac-C1 shows high enrichment in metabolic pathways such as Glycerophospholipid metabolism, Glycerolipid metabolism, and Galactose metabolism. Abnormalities in these glycolipid metabolisms can promote disease progression and prognosis^40^. UBB+Mac-C2 shows high enrichment in metabolic pathways such as Ether lipid metabolism, Citrate cycle (TCA cycle), and Butanoate metabolism^41^. In particular, the TCA cycle mediates a series of closed-loop reactions in cells to form a metabolic engine, producing various intermediate metabolites that affect the cellular environment. Macrophage polarization analysis shows that the three types of macrophage clusters tend to polarize towards the M1 type. M1 type macrophages promote inflammation at the beginning of inflammation, forming an inflammatory environment and promoting tumor growth^42^. B cell cluster-related KEGG analysis shows that TUBA1A+B cell-C0 is enriched in the IL-17 signaling pathway and Apoptosis pathway. UBE2N+B cell-C1 is enriched in the Oxidative phosphorylation pathway. Non-Aggre-B_cells-C is enriched in the Tight junction pathway. In summary, these clusters themselves affect changes in the internal molecular mechanisms of ESCC patients through corresponding pathways. In addition, communication between different immune cells also plays an important role in tumor development^43^. Our research results show that there are different intensities of cell interactions between these clusters, with clusters such as HSP90AA1-CAF-C1, TUBA1A-CAF-C0, and UBB+Mac-C2 having higher communication intensities with other subgroups. This suggests that they may jointly mediate changes in certain cancer phenotypes. Based on the maker genes of each cluster, we constructed a prognostic model at the transcriptome level and found that only the prognosis of the UBE2N+B_cell-C1 cluster showed strong heterogeneity between the training set and the validation set, but most tended to be consistent. Finally, we also revealed the sensitivity of clusters such as TUBA1A+CAF-C0, HSP90AA1+CAF-C1, VIM+CAF-C2, and TUBA1B+Mac-C0 to immunotherapy.

The Mendelian randomization results indicate a causal relationship between genes such as CTSZ, CTSC, DAD1, COLEC12, ATOX1 in the TUBA1B+Mac-C0 cluster and the occurrence of esophageal cancer. Similarly, genes like SCML4, HNRNPF, IFRD1, CSTA, ABL1 in other immune cell clusters also exhibit a causal relationship with esophageal cancer. Previous reports suggest that these genes impact the body's internal environment through various pathways. For instance, Cathepsin Z (CTSZ) and Cathepsin C (CTSC), members of the tissue proteinase family, regulate the adhesion and migration of immune cells and tumor cells^44^. CTSZ, especially macrophage-specific CTSZ, correlates with the activation of epithelial-mesenchymal transition, cell cycle characteristics, and higher infiltration levels of B cells, macrophages, neutrophils, and dendritic cells in the tumor microenvironment^45^. Tumor-secreted CTSC promotes cancer cell migration by regulating neutrophil recruitment and the formation of neutrophil extracellular traps (NET) 46. Cell apoptosis defender 1 (DAD1) is a subunit of the oligosaccharyltransferase (OST) and is a crucial negative regulator involved in programmatic cell death associated with endoplasmic reticulum^47^,^48^. Collectin subfamily member 12 (COLEC12), a member of the collectin family, acts as a pattern recognition molecule and activates the complement system through alternative pathways^49^,^50^. ATOX, a copper chaperone protein, is vital for copper transport within cells and plays a crucial role in maintaining copper homeostasis in the body. Apart from its responsibilities in copper transport, ATOX possesses functions in transcriptional regulation and body antioxidation. In cancer development, ATOX plays a significant role in regulating tumor cell migration, transcription levels, and cancer-related signaling pathways^51^. SCML4 is a transcription factor necessary for maintaining the multifunctionality of CD8+ T cells and is associated with a better prognosis in cancer patients^52^. HNRNPF, a member of the heterogeneous nuclear RNA protein family^53^, regulates RNA maturation through selective splicing, 5' capping, 3' polyadenylation, and RNA export. HNRNPF, in conjunction with proteins like VAX2 and LINC01189, promotes invasion and migration of digestive system tumor cells^54^. Interferon-related developmental regulator 1 (IFRD1) is a transcriptional co-regulator acting as a transcription modifier in the cell nucleus^55^, regulating various cell processes such as proliferation and differentiation. Recent reports suggest that IFRD1 is closely related to innate immune responses and the unfolded protein response (UPR) 56. These genes with a causal relationship with esophageal cancer may play a leading role in different cell clusters.

In conclusion, our research integrates the related information of AGG at the single-cell transcriptome and transcriptome levels, and analyzes the biological processes of different immune cells mediated by AGG. We have identified AGG-related cell clusters with prognostic characteristics and sensitivity to immunotherapy. Through Mendelian methods, we have found the Maker genes in the prognostic characteristic AGG-related cell clusters that have a causal relationship with esophageal cancer, and initially revealed the possible regulatory mechanisms for changing the expression of these genes and pathogenicity. However, due to experimental conditions and funding, we have not experimentally verified our results. In the following research, we will continue to pay attention to the biological processes mediated by AGG-related genes, and conduct related experiments under the conditions allowed by experimental funds and application conditions, to further verify our conclusions.

## Conclusion

Aggrephagy mediates and influences the alterations and interactions of various immune cells in patients with ESCC. We elucidate the roles of AGG-related clusters, such as TUBA1B+Mac-C0, VIM+CD8+T_cells-C0, UBB+Mac-C2, in mediating prognosis and immune therapy in ESCC patients. Genes causally associated with the occurrence of esophageal cancer are identified within the AGG cluster, including CTSZ, CTSC, DAD, COLEC12, ATOX1, etc., offering new evidence for clinical immune therapy. These findings underscore the significance of these gene clusters in influencing both prognosis and immune responses in the context of esophageal cancer, shedding light on potential therapeutic targets and prognostic markers.

## Figures and Tables

**Table 1:** Mendelian Randomization Validation of Maker Genes in AGG-Related Clusters

AGG_celltype	Exposure	Method	Nsnp	β	Se	Pval	Lo_ci	Up_ci	Or	Or_lci95	0r_uci95
VIM+CD8+T_cells-C0	SCML4	IVW	3	0.0007	0.0003	0.0184	0.0001	0.0013	1.0007	1.0001	1.0013
VIM+CD8+T_cells-C0	HNRNPF	IVW	3	0.0004	0.0002	0.0155	0.0001	0.0008	1.0004	1.0001	1.0008
UBB+Mac-C2	IFRD1	IVW	6	0.0004	0.0002	0.0255	0	0.0007	1.0004	1	1.0007
TUBA1B+Mac-C0	CTSZ	IVW	3	0.0006	0.0003	0.042	0	0.0012	1.0006	1	1.0012
TUBA1B+Mac-C0	CTSC	IVW	7	-0.0023	0.001	0.0268	-0.0043	-0.0003	0.9977	0.9957	0.9997
TUBA1B+Mac-C0	DAD1	IVW	2	0.0007	0.0003	0.0282	0.0001	0.0014	1.0007	1.0001	1.0014
TUBA1B+Mac-C0	COLEC12	IVW	7	0.001	0.0003	0.1095	0.0003	0.0016	1.001	1.0003	1.0016
TUBA1B+Mac-C0	C2	IVW	2	0.0005	0.0002	0.0181	0.0001	0.0009	1.0005	1.0001	1.0009
TUBA1B+Mac-C0	ATOX1	IVW	2	0.0005	0.0002	0.0596	0.0001	0.0009	1.0005	1.0001	1.0009
Non-Aggre-Mac-C1	CSTA	IVW	3	0.0016	0.0007	0.2724	0.0002	0.003	1.0016	1.0002	1.003
HSP90AA1+CAF-C1	ABL1	IVW	2	0.0004	0.0002	0.0968	0	0.0008	1.0004	1	1.0008

## Abbreviations

ESCC: Esophageal Squamous Cell Carcinoma
AGG: Aggrephagy
NMF: Non-negative Matrix Factorization
TCGA: The Cancer Genome Atlas
GEO: Gene Expression Omnibus
GSVA: Gene Set Variation Analysis
TIDE: Tumor Immune Dysfunction and Exclusion
eQTL: Expression Quantitative Trait Loci
MR: Mendelian Randomization
PCA: Principal Component Analysis
UMAP: Uniform Manifold Approximation and Projection
t-SNE: t-distributed Stochastic Neighbor Embedding
CAF: Cancer-Associated Fibroblast
MSigDB: The Molecular Signatures Database
GO: Gene Ontology
KEGG: Kyoto Encyclopedia of Genes and Genomes
SCRNA: Single-Cell RNA
COX: Cox Proportional Hazards Regression
UMI: Unique Molecular Identifiers
MSS: Microsatellite Stability
MSI: Microsatellite Instability
MSI-L: Low-frequency Microsatellite Instability
MSI-H: High Microsatellite Instability
IEU: Integrative Epidemiology Unit
GWAS: Genome-Wide Association Studies
TME: Tumor Microenvironment
IL-17: Interleukin-17
TCA: Tricarboxylic Acid
TIM-3: T cell Immunoglobulin Mucin domain 3
LAG3: Lymphocyte-activation gene 3
CTLA-4: Cytotoxic T-Lymphocyte-Associated protein 4
TIGIT: T cell Ig and ITIM domain
DEDD2: Death Effector Domain-containing protein 2
EVI2B: Ecotropic Viral Integration site 2B
TAGLN2: Transgelin 2
TOB1: Transducer of ERBB2, 1
AKT: Protein Kinase B
mTOR: Mammalian Target of Rapamycin
